# Anthropometric assessment of microtia patients’ normal ears and discussion on expander selection in auricular reconstruction surgery

**DOI:** 10.1038/s41598-022-08596-0

**Published:** 2022-03-16

**Authors:** Hefeng Sun, Pengfei Sun, Haiyue Jiang, Qinghua Yang, TongTong Li, Bo Pan

**Affiliations:** External Ear Plastic and Reconstructive Center, Department of Plastic Surgery, Plastic Surgery Hospital, Chinese Academy of Medical Sciences and Peking Union Medical College, No. 33, Ba-Da-Chu Road, Shi Jing Shan District, Beijing, 100144 China

**Keywords:** Experimental models of disease, Paediatric research, Translational research

## Abstract

The tissue expansion technique is the most suitable procedure for Chinese patients with microtia. However, it is difficult to determine whether the expanded flap is sufficient, and there are no clear or objective guidelines for determining the volume of the expander for different sizes of auricles. One hundred patients with unilateral microtia who visited our department in 2021 were randomly selected for auricular data collection using 3D scanning. The auricle length, width, projection, perimeter, and surface area were measured. Eight different volumes of expanders underwent CT and the surface areas of these expanders were measured. The surface areas of the auricles and expanders were compared and the correlation between them was explored. The average auricle parameters were calculated. The scatter plot showed a linear relationship between auricle length and auricle surface area (R^2^ = 0.9913), which demonstrated that the auricle area was approximately equal to the auricle length multiplied by 76.921. Additionally, the surface area of the expanders was measured and made into a table for selection against the surface area of the auricles. Using our equation, the auricle surface could be estimated by simply measuring the non-defective auricle length; therefore, the suitable volume of the expander could be determined.

## Introduction

For unilateral microtia, the ear on the non-defective side is of vital importance. In external ear reconstruction, plastic surgeons must perform the surgery in reference to the normal ear so that the organs on both sides are symmetrical. The more similar the two ears look, the more successful the surgery is. Thus, quantifying the parameters of the auricle has become a decisive factor in auricular reconstruction. There are many innovative ways to measure the auricle^1–3^. However, few of these are considered precise, objective, economical, time-saving, and easy to cooperate with children, who are the main group of patients with microtia who visit the hospital. Three-dimensional (3D) scanning has advantages in solving the problems mentioned above^4–7^ and has now become a routine preoperative procedure in our department for auricular data collection. The scanning device is mobile and portable. The data scanned are objective and accurate, and the subsequent data analysis is not constrained by time and location.

Tissue expanders also play an important role in auricular reconstructive surgery. In the 1950s, Neumann first used a tissue expander for auricular reconstruction^8^. Expansion-method auricular reconstruction was introduced into China in the twentieth century and has become the mainstream method ever since then^9,10^. It is considered that Asians have tighter skin in the mastoid region behind the ears; thus, the expansion method is more suitable for the Chinese because it can provide a more adequate flap area for a manmade auricle^11^. Tissue expanders are extremely elastic silicone balloons and are always overfilled to generate enough tension to expand the skin. Therefore, the final volume of an expander is highly dependent on subjective decisions rather than the specification volume. To date, however, there is no clear reference guide on how to determine the expander’s volume for different auricles. Surgeons rely on subjective feelings to decide what specification of expander to choose and the fill volume. The relationship between the expanded flap and auricle size remains vague, and it is difficult for surgeons to judge whether it is sufficient and safe to use the expanded flap to completely wrap the auricle framework.

The purpose of this study was to measure the normal ear parameters of patients with microtia among the Chinese population using 3D scanning, explore an easier way to estimate the auricle’s surface area, clarify the theoretical expansion area of different volumes of expanders, try to find the simplest correspondence between expander size and auricle size, and develop a reference guide for the selection of expanders’ specification and injection volume in auricular reconstruction.

## Results

One hundred patients (85 men and 15 women) with unilateral microtia (29 on the left side and 71 on the right side) were selected for this study. The mean age of the patients was 9.75 ± 4.2 years. The average auricle length was 58.88 ± 4.77 mm, the average auricle width was 33.15 ± 2.75 mm, the average auricle projection was 20.92 ± 2.70 mm, the average auricle perimeter was 104.67 ± 8.40 mm, and the average auricle surface area was 4502.1479 ± 633.4493 mm^2^. The data are presented in Table 1.

**Table 1:** Data summary table.

Variables	(n = 100)
Age, years	9.75 ± 4.2
**Gender**	
Male	85
Female	15
**Defective side**	
Left side	29
Right side	71
**Parameters**	
Length (mm)	58.88 ± 4.77
Width (mm)	33.15 ± 2.75
Projection (mm)	20.92 ± 2.70
Perimeter (mm)	104.67 ± 8.40
Surface area (mm^2^)	4502.1479 ± 633.4493

**Figure 1:**
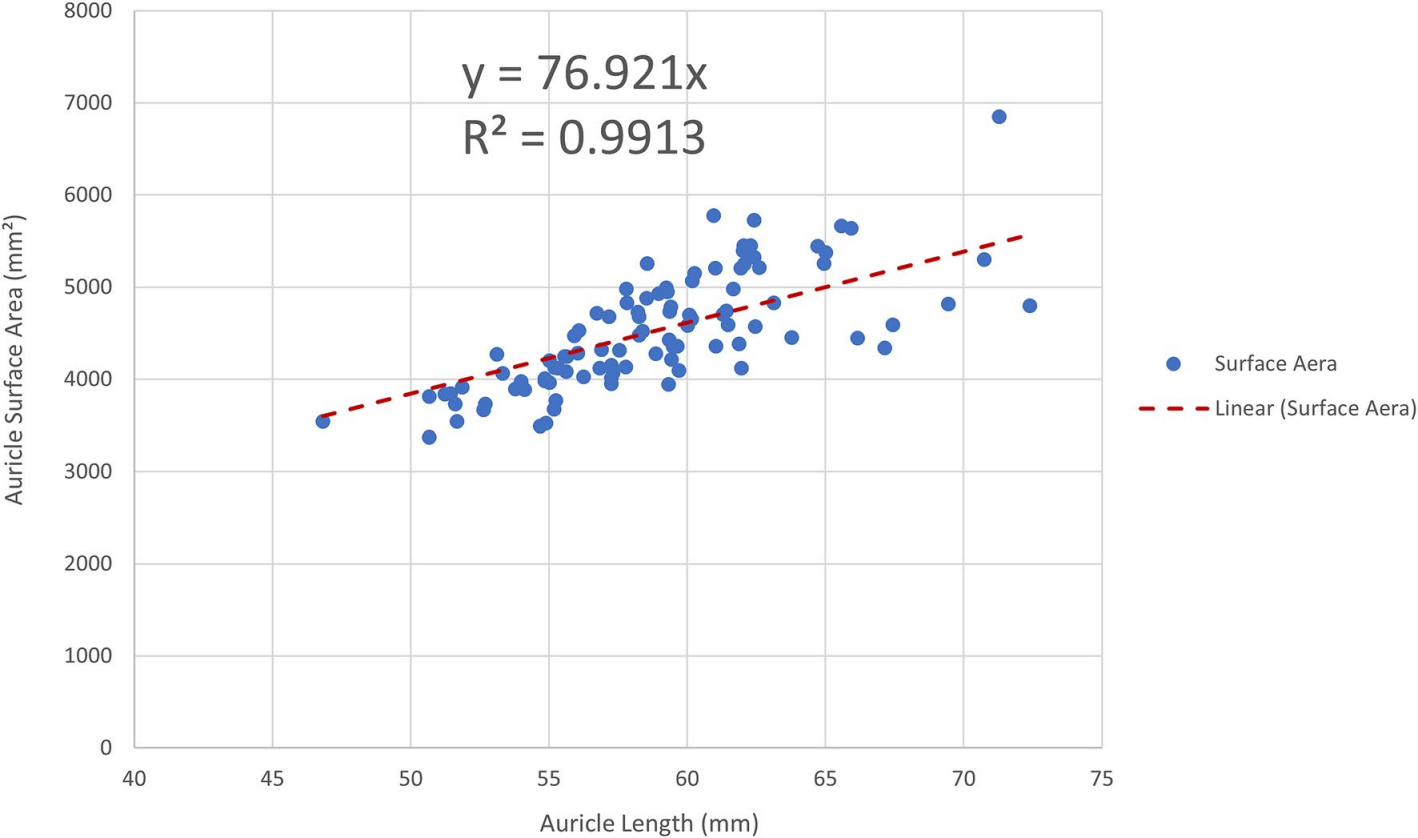
The scatter plot, linear trendline, and equation that demonstrate the relationship between auricle length and surface area. R^2^, coefficient of determination.

The scatter plot demonstrating the relationship between auricle length and surface area was as shown in Fig. 1. After excluding one set of data with significantly larger deviations (the 2nd set), a linear trend line of the scatter plot was generated. When setting the intercept as 0, the equation of the trendline is

a $$\begin{array}{*{20}c} {y = 76.921x} \\ \end{array}$$

where y is the auricle surface area and x is the auricle length (R^2^ = 0.9913).

Identical method was performed to auricle width, projection, and perimeter. When setting the intercept as 0, the equation of the trendlines are

$$y = 136.02x$$

where y is the auricle surface area and x is the auricle width (R^2^ = 0.9905);

$$y = 213.2x$$

where y is the auricle surface area and x is the auricle projection (R^2^ = 0.9785);

b $$\begin{array}{*{20}c} {y = 43.348x} \\ \end{array}$$

where y is the auricle surface area and x is the auricle perimeter (R^2^ = 0.9943).

**Figure 2:**
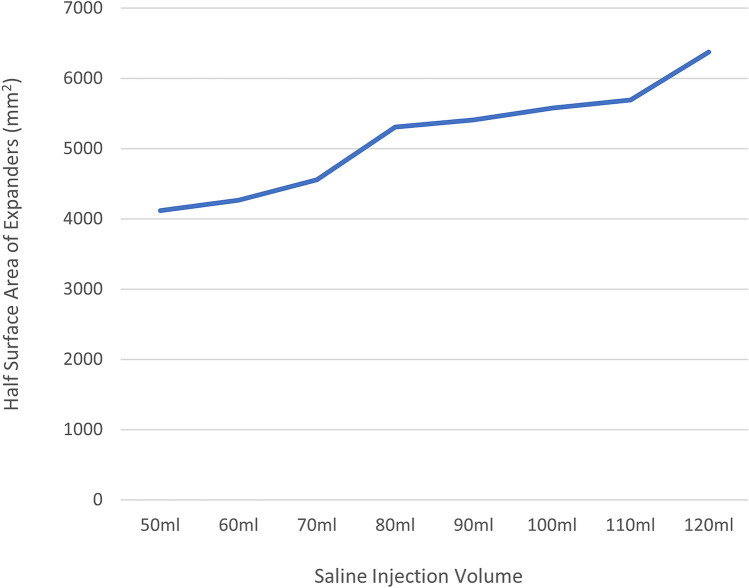
Relationship between expander’s injected volume and expander’s half surface area.

The surface areas of expanders of different sizes are listed in Table 2. Half of the area of these expanders was also measured to represent the effective expansion area. As the injection volume increased, the expander surface area increased almost linearly (Fig. 2).

**Table 2:** Surface area of the different sized expanders.

Volume injected (ml)	Full surface area (mm^2^)	Half surface area (mm^2^)
50	8227.893713	4113.947
60	8534.727777	4267.364
70	9108.005594	4554.003
80	10,616.30793	5308.154
90	10,813.37335	5406.687
100	11,158.08895	5579.044
110	11,384.74174	5692.371
120	12,752.24147	6376.121

The 50-, 60-, and 70-ml volumes were measured using a 50-ml specification expander. The remaining volumes were measured using a 100-ml specification expander.

## Discussion

Auricular reconstruction is a challenging procedure. A normal external auricle is a complex structure that has a certain degree of hardness and elasticity, with many 3D details. In 1959, Tanzer introduced the autologous rib cartilage technique^12^. Since then, the best material for auricular reconstruction has always been the costal cartilage; Brent and Nagata, and many other scholars, later modified Tanzer’s technique and developed their own methods^13–17^. To wrap the auricle framework carved with the costal cartilage, the flap amount should be sufficient. Otherwise, the tension of the flap will be too large, especially where the pressure is highest in the upper part of the helix, causing gradual ischemia, rupture of the flap, and eventual exposure of the cartilage^18–20^. However, it is difficult for surgeons to determine whether the flap is adequate. Undeniably, the area of the skin flap must be at least equal to the surface area of the contralateral auricle (non-defective side), so that the flap can exactly cover the framework. Therefore, anthropometric assessment of the normal ear is key to quantifying the surgical needs.

Many studies have attempted auricle anthropometry, but the methods vary widely. Most anthropometric methods have always been manual measurements, in which the data are subjective and inaccurate, and post-processing cannot be performed. In addition, the surface area of the auricle could not be accurately measured. Although CT before surgery can also achieve this goal, the cost is high, and patients must be exposed to unnecessary radiation. The 3D scanning method used in this study is noninvasive, convenient, portable, economical, and less time-consuming. In addition, since most patients with microtia are children, the non-noisy scanning method is easier for them to cooperate with.

**Figure 3:**
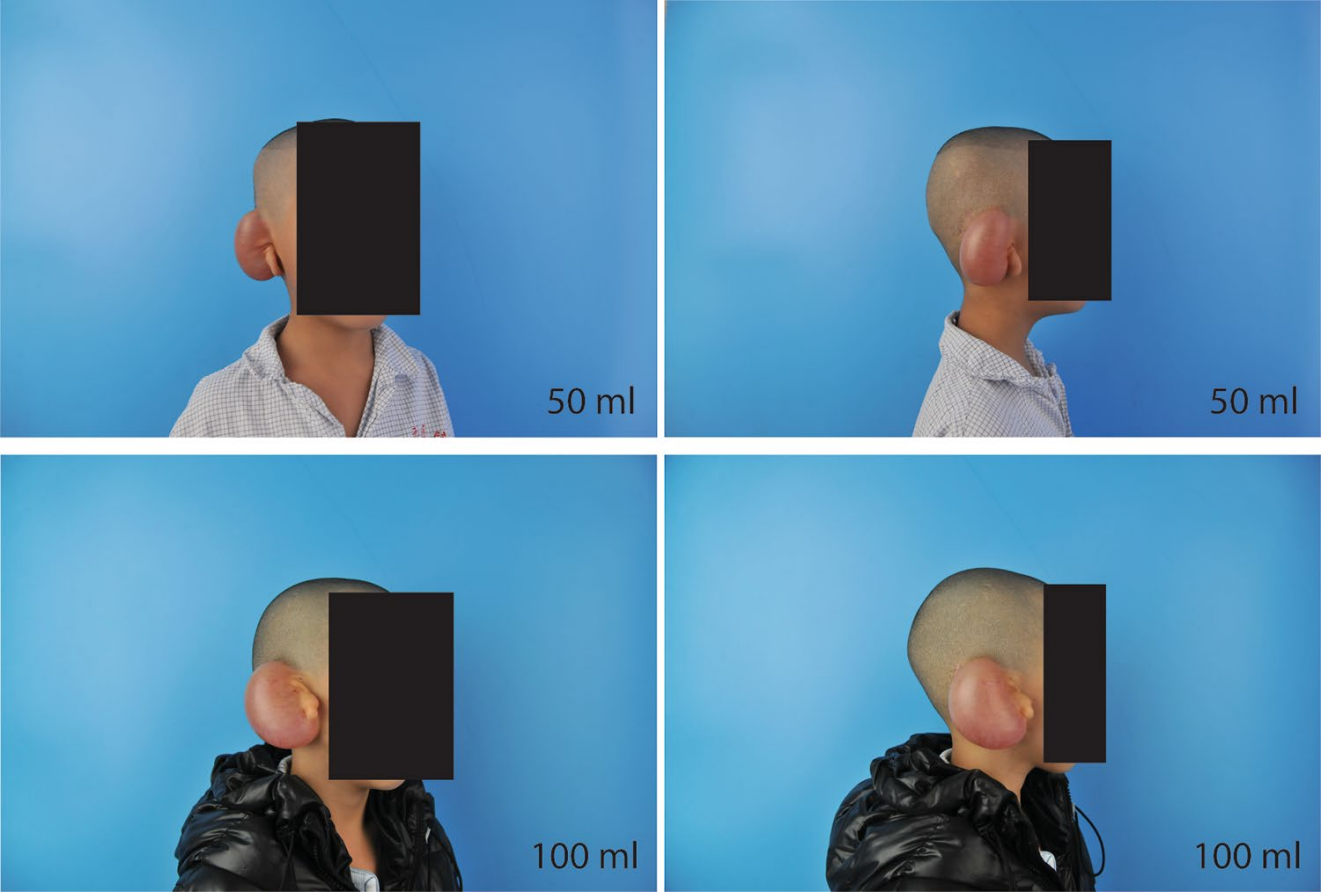
The specifications of the two expanders that are used in our clinical work. Both expanders can obtain satisfactory flaps, but there are no clear guidelines for their selection and application.

The application of expanders in auricular reconstruction is complicated and has several controversial issues. The most important issue is the quantitative relationship between the expander and the expanded flap. Some studies have scanned the expanded skin surface area using an expander under the skin^21^. However, in most cases, expanders with the same specification and volume can provide different expanded skin areas. There are two main reasons for this finding. First, the patients had different skin and soft tissue thicknesses. Patients with thicker dermis and subcutaneous fat are more difficult to expand because the tension generated by an expander of the same size is smaller in this case, and a smaller area of the expanded skin will be acquired. Second, microtia is often accompanied by varying degrees of hemifacial microsomia, which manifests as different degrees of depression of the skull in the mastoid area compared to the contralateral side. In this case, the position where the expander is buried under the skin will be relatively deep, and the part protruding from the skin level will be small; hence, the effective expansion area will be smaller. Therefore, in this study, we measured the full surface area of expanders of different sizes in vitro to prevent the influence of these factors on the results. The effective expansion area of the expanders was uniformly considered as half of the full area of the expanders. Our study showed that as the amount of saline injected increased, the expansion area increased, and the two were almost linear. Two specifications of kidney-shaped expanders (50 and 100 ml) are most commonly used in clinical practice. In most cases, both types of expanders can generate a sufficient number of flaps (Fig. 3). However, so far, there is no uniform standard for how to choose these two expanders, and opinions vary among surgeons.

To facilitate the selection of expanders in auricular reconstruction surgery, we constructed scatter plots of the measured patient’s auricle length, width, projection, perimeter and auricle area to clarify whether there was a connection between them. The trend lines of the scatter plots were automatically generated using Excel. We found that the correlation coefficient (R^2^ value) was closest to 1 only when we chose the trendline as a linear one and set the intercept to 0, which means that there is a strong linear relationship between auricle parameters and surface area. Using Eq. (a), we can roughly estimate the surface area of the auricle by simply entering the auricle length (R^2^ = 0.9913). By comparing the results to the surface area of different expanders (Table 2), we can clarify which expander should be selected to produce a sufficient area to completely wrap the auricle framework. However, considering that the elastic shrinkage of the expanded skin and the flap tension cannot be too large after surgery, a slightly larger (at least 10 ml larger) expander should be selected. Although Eq. (b) has a slightly higher R^2^ (R^2^ = 0.9943), which suggests there is a stronger linear relationship between auricle perimeter and auricle area, it is much harder and more inaccurate to measure the auricle perimeter in clinical work without 3D scanning or any other digital devices. In contrast, auricle length is much easier to measure without large error. Surgeons could get the auricle length in a few seconds by simply using a flexible tape. Therefore, estimating auricle area by measuring auricle length would be the more preferable way.

Combining our results with our surgical experience, we recommend that it is safe and has a satisfactory surgical outcome of completely wrapping the auricular framework with an expanded flap for patients whose auricle length is less than 58 mm. In this case, the auricular surface area is approximately 4461.42 mm^2^ by Eq. (a), and a 50 ml expander overfilled with 70 ml of saline could be selected according to Table 2. For patients with auricle lengths greater than 58 mm, we do not recommend completely wrapping the auricle framework with an expanded skin flap. An oversized framework causes greater pressure on the skin flap at the helix, leading to avascular necrosis and rupture of the skin flap, followed by an exposed framework. In addition, the hairless skin on the affected side of the microtia is limited, and an excessively large expanded skin flap will inevitably expand the scalp too much, causing the reconstructed auricle to have too much hair and affect its appearance. For patients with auricle lengths greater than 58 mm, we recommend wrapping the front of the auricular framework and most of the helix with an expanded skin flap and then lifting the temporal muscle fascia flap behind the framework and grafting skin on its surface. This method not only relieved the tension of the skin flap but also made a higher auricle and a clearer and more apparent ear-cranial groove (Table 3).

**Table 3:** Our experience-based guidelines regarding the use of the expanded flap in auricular reconstruction.

Auricle length (mm)	Recommend method of surgery
≤ 58	Completely wrap the ear framework with an expanded flap
> 58	Partially wrap the ear framework with an expanded flap, combined with skin grafting

In conclusion, 3D scanning is non-invasive, easier for children to cooperate with, more accurate for data measurement, and less time-consuming for auricular data collection. The auricle length and surface area had a roughly linear relationship. The equation is as follows:

$$\begin{array}{*{20}c} {y = 76.921x} \\ \end{array}$$

where y is the auricle surface area and x is the auricle length (R^2^ = 0.9913). Simply measuring the length of the auricle could estimate the area of the auricle and guide the choice of the expander volume.

## Methods

This study was reported in accordance with the Strengthening the Reporting of Observational studies in Epidemiology (STROBE) statement^22^.

### Ethics approval and consent to participate

Patients and their families provided written informed consent for participation in the study. The patients/legal guardians agreed to use their images for publication of this article and signed the informed consent. All procedures performed in studies involving human participants were in accordance with the ethical standards of the institutional and/or national research committee and with the 1964 Helsinki Declaration and its later amendments or comparable ethical standards. This study was approved by the Medical Ethics Committee of Plastic Surgery Hospital, Chinese Academy of Medical Sciences (file no. 2020-186).

### Participants

For auricle anthropometry, 100 patients with microtia who visited our department in 2021 were randomly selected for the study before auricular reconstruction surgery. All participants in this study were patients with unilateral microtia patients aged 5–10 years. Other exclusion criteria were as follows: (1) patients with any auricular deformity of the healthy side, such as prominent ears, Stahl’s ears, and cryptotia; (2) patients with severe hemifacial microsomia; (3) patients with auricular trauma and/or surgery history of the healthy side; and (4) extremely uncooperative patients.

### Measurements

#### Auricle anthropometry

**Figure 4:**
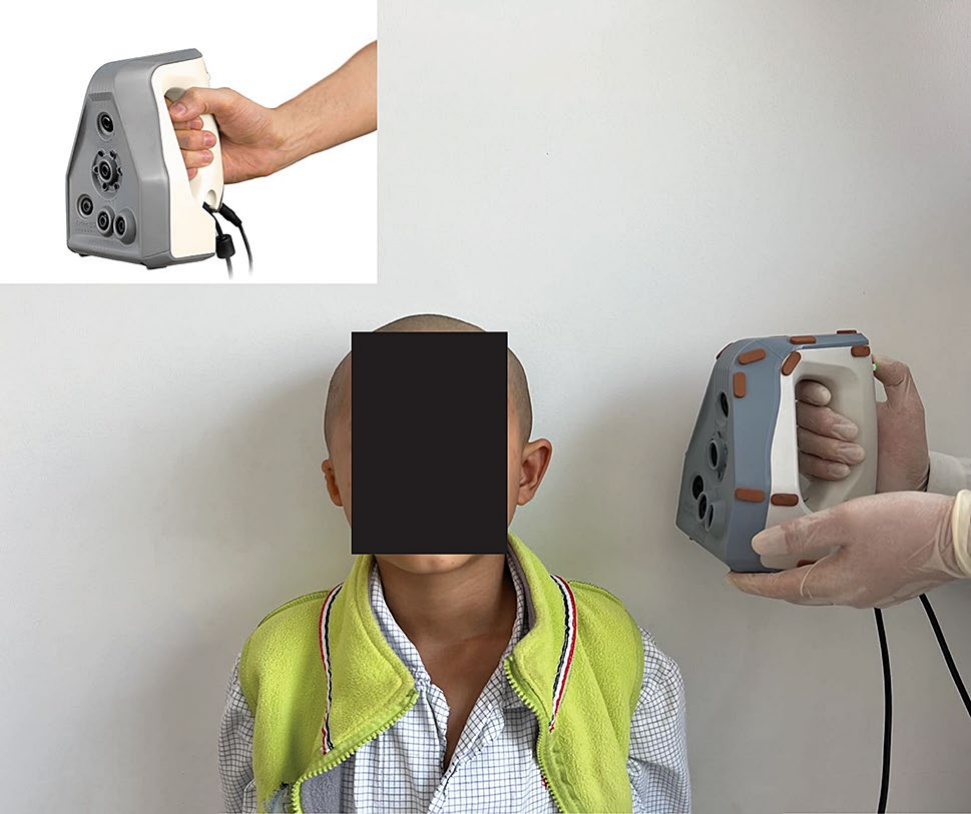
Method and devices of auricle scanning.

**Figure 5:**
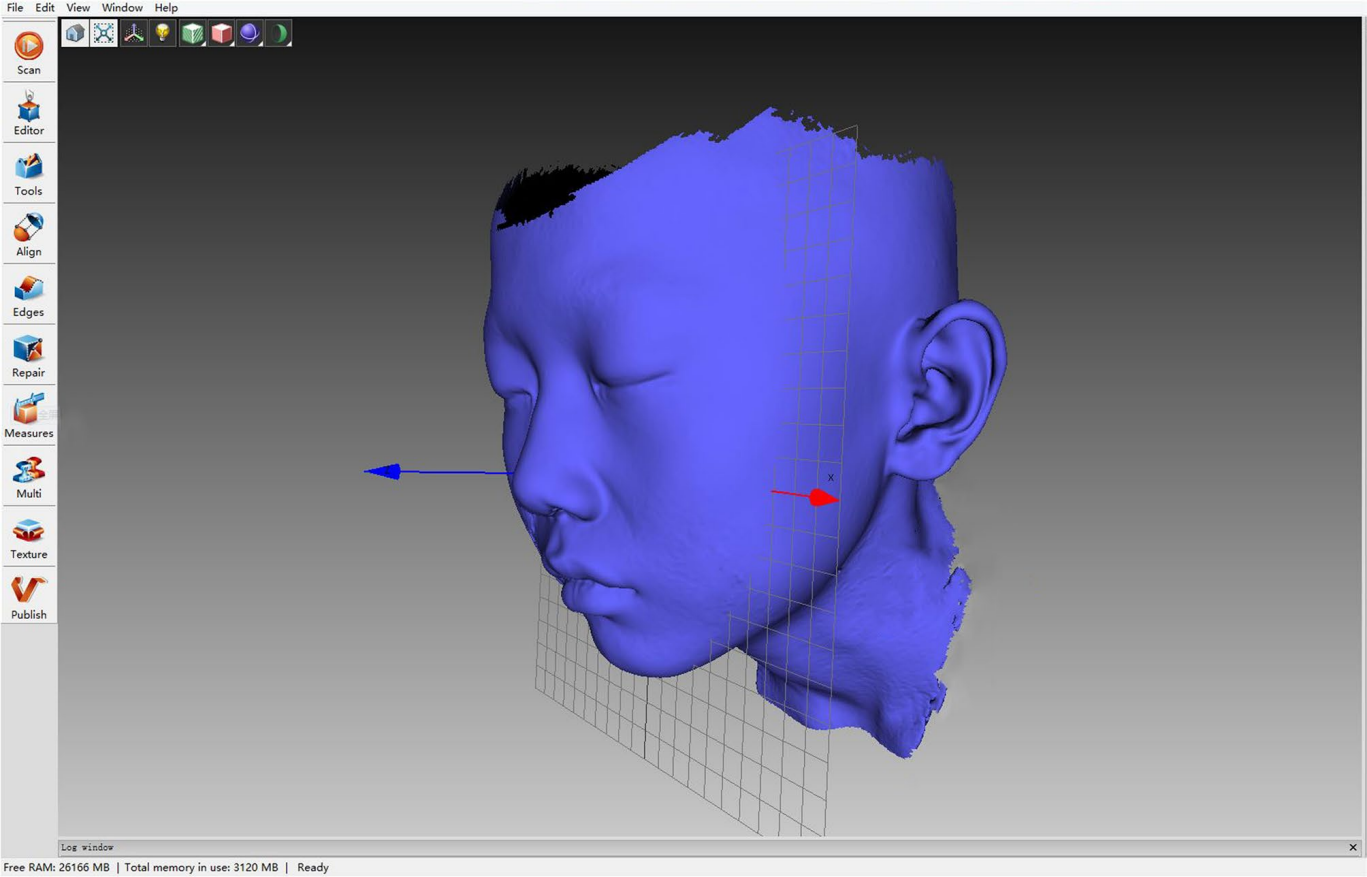
Scanning data were collected and synchronized into STL files by Artec Studio 10.

**Figure 6:**
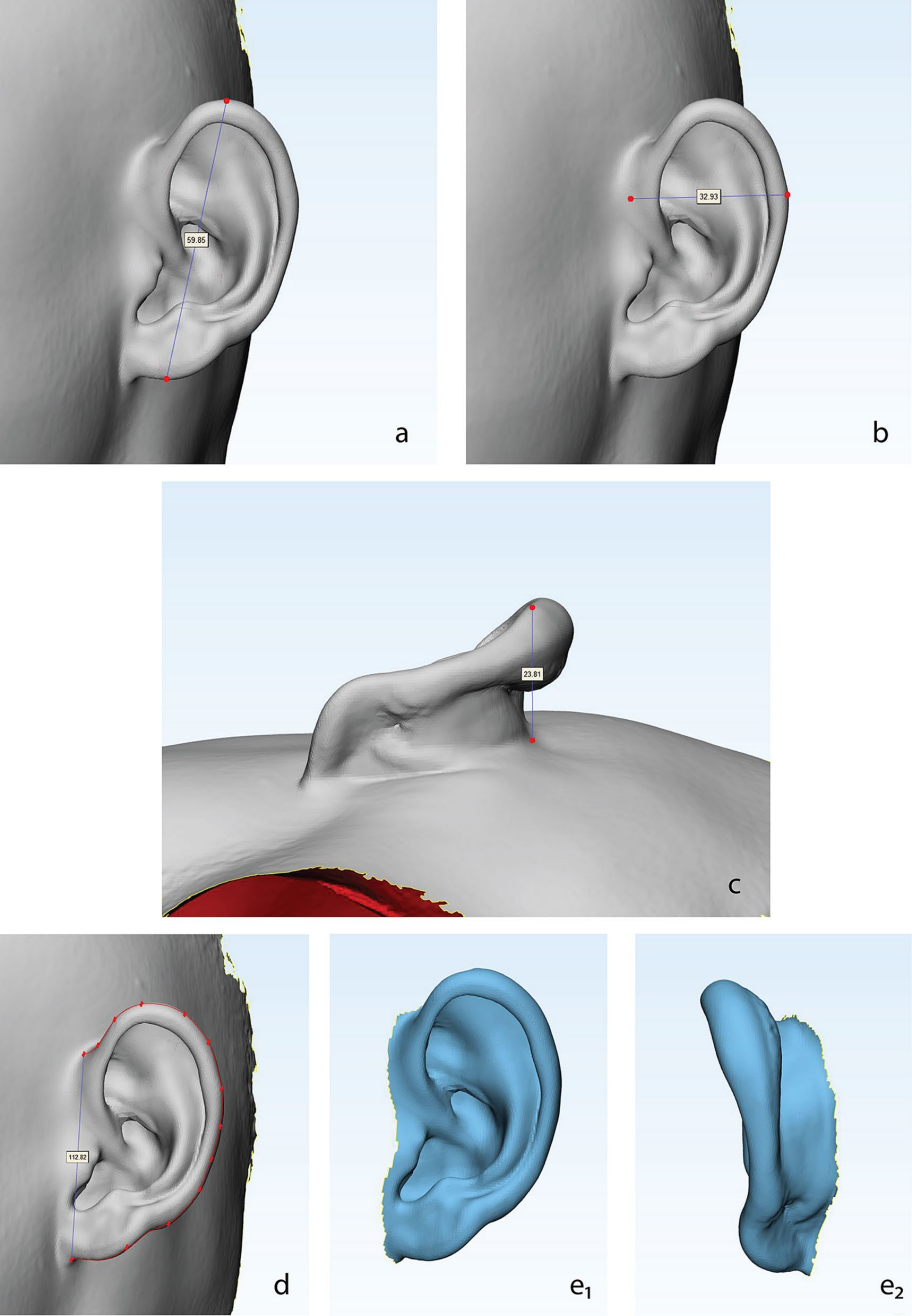
Five factors of the normal auricle measured in this study, including ear length (**a**), ear width (**b**), ear projection (**c**), ear perimeter (**d**), and ear surface area (front **e**1 and back **e**2).

Patients were asked to remove all types of earrings or headwear and have a haircut to fully expose the bilateral ears. The heads of all selected patients (including the entire face and bilateral ears) were scanned using an Artec Space Spider 3D Scanner (Artec 3D, Redmond, USA) (Fig. 4). The scanning data were synthesized into STL files using Artec Studio 10 (Artec 3D, Redmond, USA) and then imported into 3-matic Research 9.0 (Materialise NV, Leuven, Belgium), where all the measurements were performed (Fig. 5). The following five factors of the normal ear of the enrolled microtia patients were measured (Fig. 6).

- Ear length: Perpendicular distance from the highest point on the border of the helix to the lowest point of the lobule.
- Ear width: Perpendicular distance from the attachment point of the auricle and face to the outermost point of the helix.
- Ear projection: Perpendicular distance from the outermost point on the helix to the cranial plane behind the ear.
- Ear perimeter: Curve length of the auricle, which starts from the attachment point of the auricle and skin of the face to the attachment point of the lobule and face.
- Ear surface area: Surface area of the whole auricle, measured from the perpendicular line of the helix foot and tragus notch to the ear-cranial groove.

All the data were collected and entered into a table. The length and surface area of the auricles were plotted on a scatter plot, and a linear regression equation was calculated.

#### Expander volume measurement

**Figure 7:**
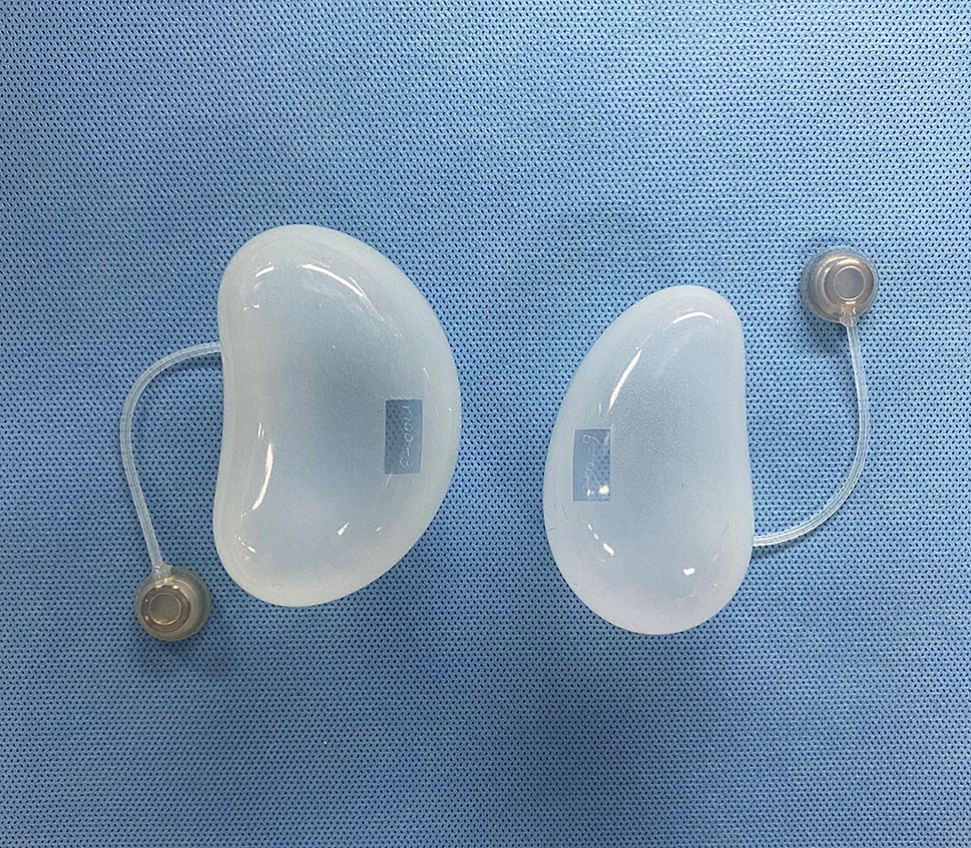
The specifications of the two tissue expanders that are most commonly used in auricular reconstruction. Left 100 ml, right 50 ml.

For expander volume measurement, two specifications (50 ml and 100 ml) of kidney-shaped soft tissue expanders (Jiusheng Medical Supply, Yuyao, China), which are most commonly applied in expansion auricular reconstructive surgery, were used in this study (Fig. 7).

**Figure 8:**
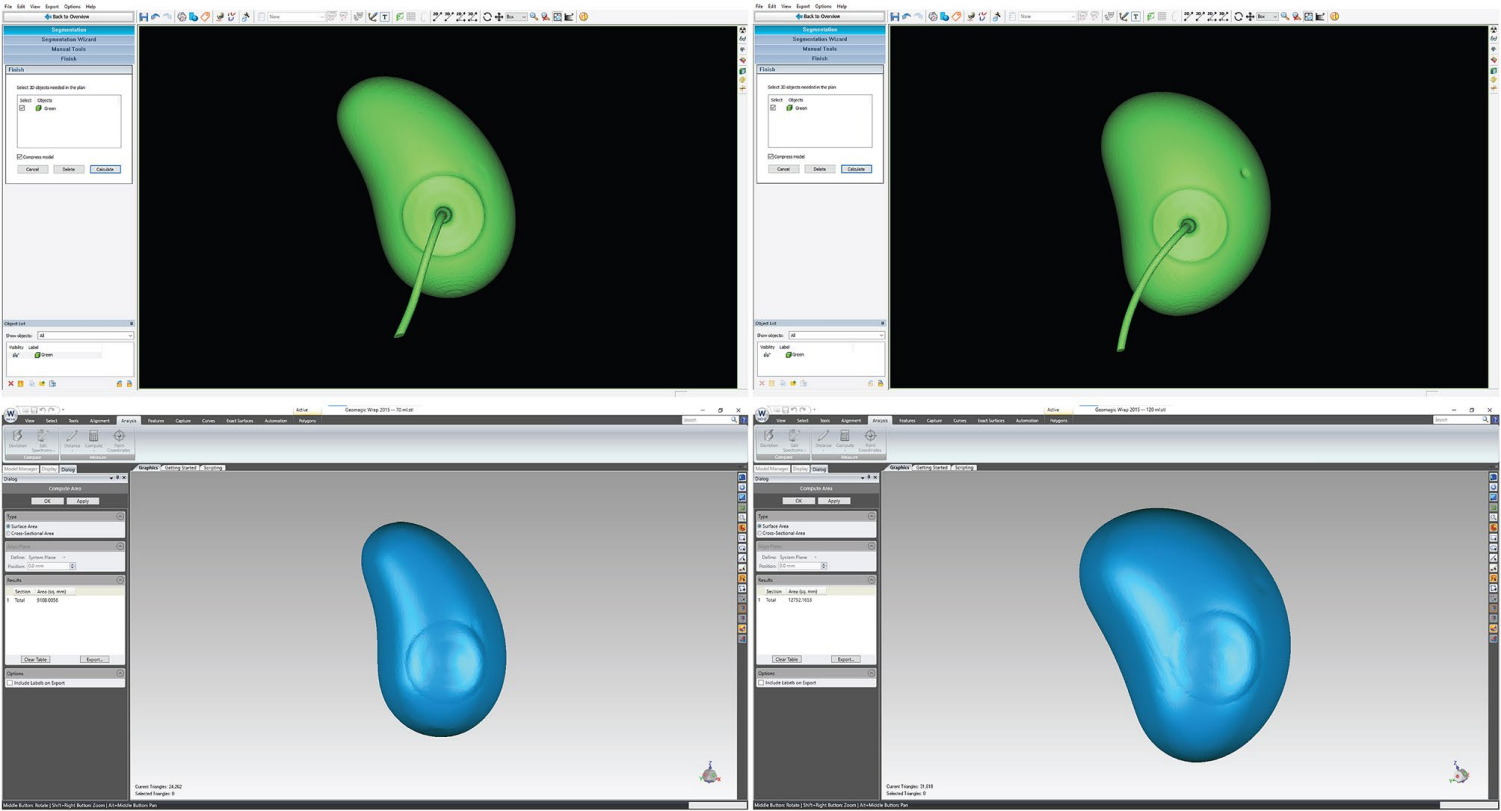
DICOM data of the expanders were processed in ProPlan (above), and the surface area was measured in Geomagic Wrap (below).

Three 50 ml expanders were injected with 50 ml, 60 ml, and 70 ml of saline. Five 100 ml expanders were injected with 80, 90, 100, 110, and 120 ml of saline. All eight expanders underwent computed tomography (CT) after injection (Brilliance CT 64 slice, Philips Medical Systems, Cleveland, OH; tube voltage, 120 kVp; tube current, 220 mAs; collimation, 0.6 mm; pitch, 0.8; rotation time, 0.75 s; matrix, 512 512; and field of view, 350 mm). DICOM data were then acquired and imported to ProPlan CMF 3.0 (Materialise NV, Leuven, Belgium), where the injection hose and injection pots were removed manually and STL files of expanders were created. All STL files were then imported into Geomagic Wrap 2015 (3D Systems Inc., Rock Hill, USA), and the surface area of the expanders was measured automatically using the software (Fig. 8).

### Data analysis

All participants’ auricle length, width, projection, perimeter, and surface area were collected and put in a table. Auricle length, width, projection, and perimeter were taken as independent variables respectively; auricle surface area was taken as dependent variables and their relationships were drawn with scatterplots. Linear trend lines were generated along with their equations and their correlation coefficient (R^2^ value).

### Statistical analysis

All anthropometric data were subjected to statistical analysis using SPSS 25 (IBM Corp., NY, USA). The trend line and linear equations were performed using Microsoft 365 Excel.

## Supplementary Information

Supplementary Information.

## Data Availability

The data that support the findings of this study are available in the Supplementary Information of this article.
