# Supplementary material for: Anthropometric assessment of microtia patients’ normal ears and discussion on expander selection in auricular reconstruction surgery
Source: Sci Rep. 2022 Mar 16;12:4521. doi: 10.1038/s41598-022-08596-0 (PMC8927408; doi:10.1038/s41598-022-08596-0)
Supplement: Supplementary file 1 — Supplementary Information. [file 41598_2022_8596_MOESM1_ESM.docx]

Supplementary Information: Measurement Data of All Patients

| NO. | Side | Length (mm) | Width (mm) | Height (mm) | Perimeter (mm) | Surface Aera (mm^2^) |
| --- | --- | --- | --- | --- | --- | --- |
| 1 | Right | 67.43 | 30.18 | 16.54 | 106.43 | 4589.0245 |
| 2 | Right | 65.38 | 27.91 | 20.08 | 119.14 | 3132.2328 |
| 3 | Right | 51.60 | 29.94 | 20.35 | 96.76 | 3728.8665 |
| 4 | Right | 58.87 | 34.55 | 22.31 | 106.79 | 4277.3521 |
| 5 | Right | 69.44 | 34.00 | 24.05 | 113.65 | 4815.8668 |
| 6 | Left | 61.88 | 29.72 | 23.50 | 88.84 | 4381.0549 |
| 7 | Right | 53.11 | 35.16 | 22.21 | 100.86 | 4267.4699 |
| 8 | Right | 51.45 | 31.39 | 22.16 | 96.53 | 3841.7515 |
| 9 | Left | 62.47 | 33.98 | 22.69 | 106.92 | 4574.1849 |
| 10 | Right | 57.54 | 34.27 | 23.27 | 103.27 | 4314.7788 |
| 11 | Left | 50.67 | 27.51 | 19.70 | 92.37 | 3374.5274 |
| 12 |  | 56.90 | 29.60 | 19.44 | 101.76 | 4322.8909 |
| 13 | Right | 61.96 | 28.65 | 23.12 | 101.21 | 4120.4258 |
| 14 | Right | 57.16 | 30.17 | 25.67 | 100.82 | 4678.4931 |
| 15 | Right | 58.52 | 34.24 | 25.72 | 114.22 | 4877.6402 |
| 16 | Right | 54.90 | 32.88 | 14.70 | 93.97 | 3526.0189 |
| 17 | Left | 72.39 | 37.79 | 23.25 | 121.72 | 4797.3370 |
| 18 | Left | 52.63 | 30.49 | 18.82 | 91.94 | 3667.5917 |
| 19 | Right | 59.40 | 34.06 | 20.72 | 111.76 | 4787.4737 |
| 20 | Right | 57.25 | 29.23 | 17.27 | 95.38 | 4152.4872 |
| 21 | Right | 54.68 | 26.50 | 20.47 | 96.40 | 3490.4895 |
| 22 | Left | 62.06 | 35.04 | 22.88 | 112.56 | 5248.4046 |
| 23 | Right | 63.78 | 32.07 | 18.75 | 105.36 | 4452.8728 |
| 24 | Right | 57.80 | 35.37 | 23.74 | 112.23 | 4981.1124 |
| 25 | Right | 58.25 | 32.80 | 20.69 | 100.87 | 4479.4804 |
| 26 | Right | 64.71 | 35.73 | 21.04 | 112.89 | 5442.3562 |
| 27 | Right | 62.05 | 35.29 | 25.00 | 108.16 | 5450.4406 |
| 28 | Right | 58.55 | 34.20 | 21.47 | 109.36 | 5258.0344 |
| 29 | Left | 56.08 | 32.80 | 26.61 | 98.48 | 4530.5397 |
| 30 | Left | 57.32 | 33.92 | 17.98 | 99.21 | 4067.4066 |
| 31 | Right | 58.38 | 33.73 | 21.24 | 102.86 | 4523.6220 |
| 32 | Left | 67.15 | 31.39 | 26.30 | 98.62 | 4341.3974 |
| 33 | Left | 56.83 | 32.39 | 17.53 | 102.10 | 4117.2624 |
| 34 | Left | 62.60 | 34.59 | 22.44 | 115.72 | 5213.3476 |
| 35 | Left | 54.11 | 33.39 | 18.40 | 94.50 | 3890.1247 |
| 36 | Right | 66.16 | 32.98 | 14.89 | 104.62 | 4443.9941 |
| 37 | Right | 61.93 | 34.44 | 24.28 | 110.84 | 5203.0481 |
| 38 | Right | 55.01 | 34.63 | 20.99 | 101.94 | 3965.4077 |
| 39 | Right | 60.07 | 32.55 | 18.86 | 108.82 | 4695.1777 |
| 40 | Right | 56.73 | 34.62 | 18.77 | 103.32 | 4715.8716 |
| 41 | Right | 56.04 | 35.23 | 22.83 | 102.20 | 4284.9139 |
| 42 | Right | 55.19 | 31.80 | 18.82 | 91.89 | 3671.4409 |
| 43 | Right | 61.66 | 36.38 | 21.32 | 113.30 | 4977.0681 |
| 44 | Right | 62.41 | 38.24 | 22.56 | 120.85 | 5724.1113 |
| 45 | Right | 59.42 | 34.24 | 21.55 | 105.05 | 4215.2573 |
| 46 | Right | 61.48 | 34.45 | 21.39 | 105.47 | 4590.8943 |
| 47 | Right | 59.50 | 34.43 | 18.27 | 99.48 | 4353.0278 |
| 48 | Right | 46.82 | 29.43 | 21.33 | 90.55 | 3545.3908 |
| 49 | Left | 61.28 | 29.82 | 17.48 | 104.80 | 4703.6600 |
| 50 | Right | 59.32 | 30.60 | 21.14 | 97.48 | 3941.2692 |
| 51 | Right | 55.21 | 30.04 | 17.45 | 97.39 | 4128.4589 |
| 52 | Right | 61.01 | 35.78 | 20.05 | 112.99 | 5205.6787 |
| 53 | Right | 63.12 | 36.25 | 24.22 | 106.03 | 4828.5493 |
| 54 | Right | 62.41 | 36.16 | 24.32 | 116.08 | 5322.4073 |
| 55 | Right | 55.01 | 29.80 | 19.69 | 101.16 | 4201.2397 |
| 56 | Right | 59.69 | 32.30 | 20.09 | 106.40 | 4093.8465 |
| 57 | Left | 57.78 | 33.02 | 19.32 | 99.56 | 4131.6302 |
| 58 | Right | 71.28 | 42.90 | 20.99 | 131.77 | 6849.9914 |
| 59 | Right | 60.27 | 36.42 | 22.01 | 114.88 | 5151.4412 |
| 60 | Right | 59.37 | 33.28 | 20.83 | 104.71 | 4733.0721 |
| 61 | Right | 55.57 | 33.51 | 17.43 | 96.82 | 4248.5731 |
| 62 | Right | 55.24 | 32.79 | 18.45 | 98.11 | 3766.5320 |
| 63 | Right | 53.33 | 28.79 | 17.30 | 93.81 | 4060.6947 |
| 64 | Right | 62.30 | 34.98 | 22.29 | 112.57 | 5448.2867 |
| 65 | Left | 62.03 | 33.21 | 21.87 | 113.12 | 5391.0063 |
| 66 | Left | 58.97 | 32.68 | 20.12 | 109.39 | 4931.2592 |
| 67 | Right | 55.63 | 34.52 | 23.36 | 99.01 | 4080.7345 |
| 68 | Left | 54.00 | 30.44 | 21.72 | 96.58 | 3974.7216 |
| 69 | Left | 61.41 | 34.95 | 23.20 | 113.03 | 4740.9456 |
| 70 | Left | 70.73 | 35.57 | 21.61 | 115.32 | 5297.4226 |
| 71 | Left | 65.01 | 33.20 | 23.53 | 109.72 | 5373.2174 |
| 72 | Right | 60.95 | 33.41 | 22.28 | 115.39 | 5774.4880 |
| 73 | Right | 64.94 | 38.71 | 18.35 | 115.14 | 5258.9352 |
| 74 | Right | 51.24 | 30.79 | 21.16 | 92.18 | 3836.9483 |
| 75 | Left | 60.16 | 32.57 | 18.05 | 106.81 | 4656.5632 |
| 76 | Left | 55.65 | 30.01 | 21.31 | 99.66 | 4245.7618 |
| 77 | Right | 59.24 | 36.13 | 24.36 | 114.33 | 4990.5487 |
| 78 | Right | 59.28 | 37.61 | 23.97 | 112.69 | 4950.9119 |
| 79 | Right | 51.67 | 29.78 | 18.39 | 87.30 | 3543.2221 |
| 80 | Right | 53.78 | 30.65 | 19.36 | 97.30 | 3896.1964 |
| 81 | Right | 50.66 | 32.60 | 21.82 | 93.65 | 3810.1540 |
| 82 | Right | 58.26 | 32.77 | 20.62 | 108.97 | 4680.8362 |
| 83 | Right | 54.85 | 33.82 | 15.65 | 104.12 | 3980.9307 |
| 84 | Right | 65.94 | 37.31 | 24.02 | 114.79 | 5641.1231 |
| 85 | Right | 65.58 | 36.96 | 24.19 | 119.61 | 5660.8391 |
| 86 | Right | 57.25 | 30.12 | 21.61 | 93.63 | 4014.8794 |
| 87 | Right | 56.26 | 31.77 | 19.10 | 99.48 | 4026.5125 |
| 88 | Left | 57.82 | 33.61 | 22.78 | 100.57 | 4826.2132 |
| 89 | Right | 60.18 | 34.16 | 24.16 | 114.02 | 5066.3221 |
| 90 | Left | 54.85 | 30.25 | 20.87 | 96.82 | 4008.4578 |
| 91 | Right | 60.01 | 33.31 | 18.72 | 109.72 | 4583.3058 |
| 92 | Right | 58.22 | 35.99 | 16.74 | 108.94 | 4726.5805 |
| 93 | Left | 55.91 | 37.73 | 27.33 | 110.20 | 4472.6234 |
| 94 | Right | 59.35 | 35.16 | 15.30 | 107.31 | 4424.5574 |
| 95 | Right | 52.70 | 30.71 | 18.55 | 97.65 | 3732.2085 |
| 96 | Right | 61.04 | 32.85 | 20.01 | 104.05 | 4358.4498 |
| 97 | Left | 55.33 | 30.54 | 23.09 | 95.37 | 4118.7436 |
| 98 | Right | 57.25 | 30.07 | 15.56 | 102.86 | 3950.6856 |
| 99 | Left | 51.85 | 34.9 | 20.47 | 96.22 | 3912.2860 |
| 100 | Left | 59.63 | 31.53 | 19.77 | 105.73 | 4360.8991 |
| Mean |  | 58.88 ± 4.77 | 33.15 ± 2.75 | 20.92 ± 2.70 | 104.67 ± 8.40 | 4502.1479 ± 633.4493 |
